# A three-dimensional immune-oncology model for studying *in vitro* primary human NK cell cytotoxic activity

**DOI:** 10.1371/journal.pone.0264366

**Published:** 2022-03-21

**Authors:** Nontaphat Thongsin, Methichit Wattanapanitch

**Affiliations:** 1 Research Department, Siriraj Center for Regenerative Medicine, Faculty of Medicine Siriraj Hospital, Mahidol University, Bangkok, Thailand; 2 Department of Immunology, Faculty of Medicine Siriraj Hospital, Mahidol University, Bangkok, Thailand; Children’s National Hospital, UNITED STATES

## Abstract

Immunotherapy has emerged as a promising therapeutic approach for treating several forms of cancer. Adoptive cell transfer of immune cells, such as natural killer (NK) cells, provides a powerful therapeutic potential against tumor cells. In the past decades, two-dimensional (2D) tumor models have been used to investigate the effectiveness of immune cell killing. However, the 2D tumor models exhibit less structural complexity and cannot recapitulate the physiological condition of the tumor microenvironment. Thus, the effectiveness of immune cells against tumor cells using these models cannot fully be translated to clinical studies. In order to gain a deeper insight into immune cell-tumor interaction, more physiologically relevant *in vivo*-like three-dimensional (3D) tumor models have been developed. These 3D tumor models can mimic the dynamic cellular activities, making them a much closer representation of the *in vivo* tumor profiles. Here, we describe a simple and effective protocol to study the cytotoxic activity of primary human NK cells toward the 3D tumor spheroids. Our protocol includes isolation and expansion of human NK cells, labeling and formation of tumor spheroids, co-culture of NK cells and tumor spheroids, and evaluation of cytotoxic activity using a confocal microscope. This protocol is also applicable to other types of tumors and immune cells.

## Introduction

Cancer immunotherapy is a treatment strategy that aims to manipulate immune cells to recognize and attack tumor cells either alone or in combination with other standard treatments such as surgery, chemotherapy, and radiation therapy [1]. Adoptive cell transfer (ACT) is emerging as one of the practical therapeutic approaches for cancer therapy. Several types of immune cells, such as T cells, B cells, natural killer (NK) cells, dendritic cells (DC), and macrophages, have been used in pre-clinical and clinical studies [2]. NK cells, which are the subset of lymphocytes that provide the first-line defense against infected and tumor cells, have been investigated for their potential to induce cytotoxicity in tumor cells [3, 4]. Unlike T cells, NK cells can recognize tumor cells in a human leukocyte antigen (HLA)-independent manner. Data from several clinical studies demonstrated great therapeutic outcomes without graft-versus-host disease (GvHD) after administering allogeneic NK cells to patients with relapsed acute myeloid leukemia or several solid tumors such as renal cell carcinoma, ovarian cancer, and non-small lung cancer [5–7]. Therefore, allogeneic NK cells represent an alternative therapeutic cell source as off-the-shelf cell products [8]. Moreover, genetic modification can be performed to improve cytotoxicity, specificity, and persistence of the NK cell products [9, 10]. For these reasons, validation of NK cell effector functions using an appropriate tumor model is required.

Previously, the *in vitro* 2D tumor models have been widely used to determine the cytotoxicity of human immune cells. However, these models do not recapitulate the complexity of the tumor environment due to the lack of cellular interaction and the interaction with extracellular matrices [11, 12]. To overcome these limitations, a more relevant 3D tumor model to study the efficacy of human immune cells has been developed. The first 3D tumor model has been shown to mimic the physiological properties of the cells in terms of topographical and mechanical forces, rendering the cellular responses to stimuli differently [13–16]. More recently, several studies indicated that the 3D tumor model altered various cellular activities, including morphology, signal transduction, gene and protein expression, and drug sensitivity when compared to the 2D models [17–19]. Notably, the structural complexity of tumor spheroids can prevent the infiltration of immune cells, making them a versatile *in vivo*-like model [20, 21]. Thus, the use of 3D tumor spheroids provides a reliable tool for assessing the therapeutic efficacy of immune cells. Here, we describe a simple protocol for generating 3D tumor spheroids to evaluate the *in vitro* cytotoxicity of primary human NK cells (PB-NK) against the cholangiocarcinoma cell line (KKU213A) [22]. We also provide a step-by-step protocol for the cytotoxicity assay, including isolation and expansion of NK cells, labeling and formation of tumor spheroids, and evaluation of the cytotoxic activity of NK cells against tumor spheroids. This protocol can be easily adapted for other tumor and immune cells, including primary and genetically modified human immune cells.

## Materials and methods

The protocol described in this peer-reviews article is published on protocols.io, dx.doi.org/10.17504/protocols.io.b2ufqetn and is included for printing as S1 File with this article.

### Ethics statement

All procedures complied with ethical regulations for research in humans. This study was approved by the Institutional Review Board (IRB), Faculty of Medicine Siriraj Hospital, Mahidol University (Si 320/2021). All patients were informed about using their samples and provided with a participant information sheet and signed the informed consent.

## Results

**Fig 1:**
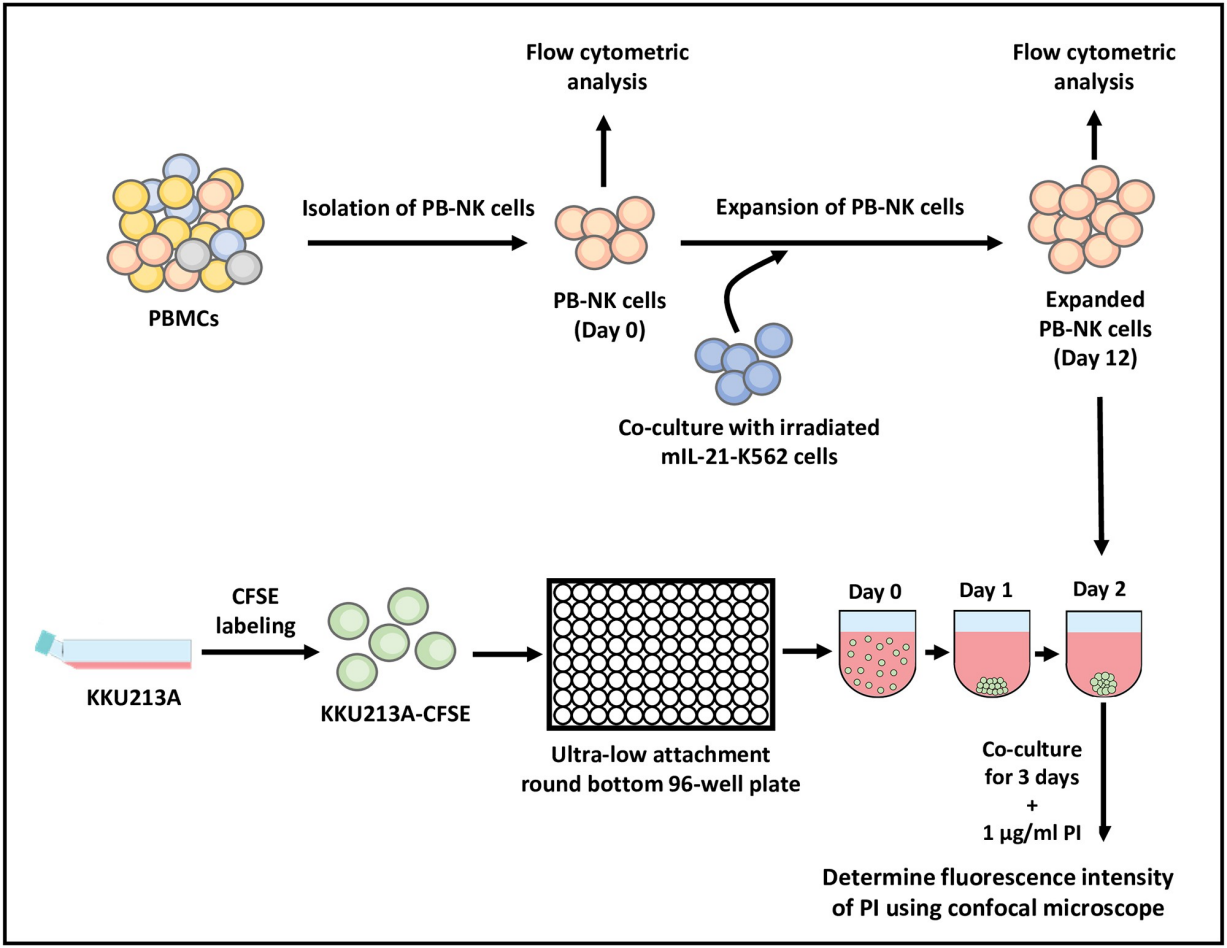
Schematic diagram of cytotoxicity assay using PB-NK cells toward the 3D tumor spheroids.

In this protocol, we utilized the 3D tumor spheroid model to evaluate the cytotoxic activity of primary human NK cells from two healthy donors (Fig 1).

The PB-NK cells were isolated using magnetic beads and expanded using a genetically modified mIL-21-K562 cell line. The purity of the isolated and expanded PB-NK cells was determined by flow cytometric analysis. The 3D tumor spheroids were formed using the CFSE-labeled KKU213A cell line and cultured in the medium containing 2.5% Matrigel on an ultra-low attachment round-bottom 96-well plate for two days. The spheroids were co-cultured with PB-NK cells supplemented with propidium iodide (PI) for three days. The cytotoxic activity of NK cells was evaluated using mean fluorescence intensity (MFI) of PI, which stained positive on dead cells using a confocal microscope.

**Fig 2:**
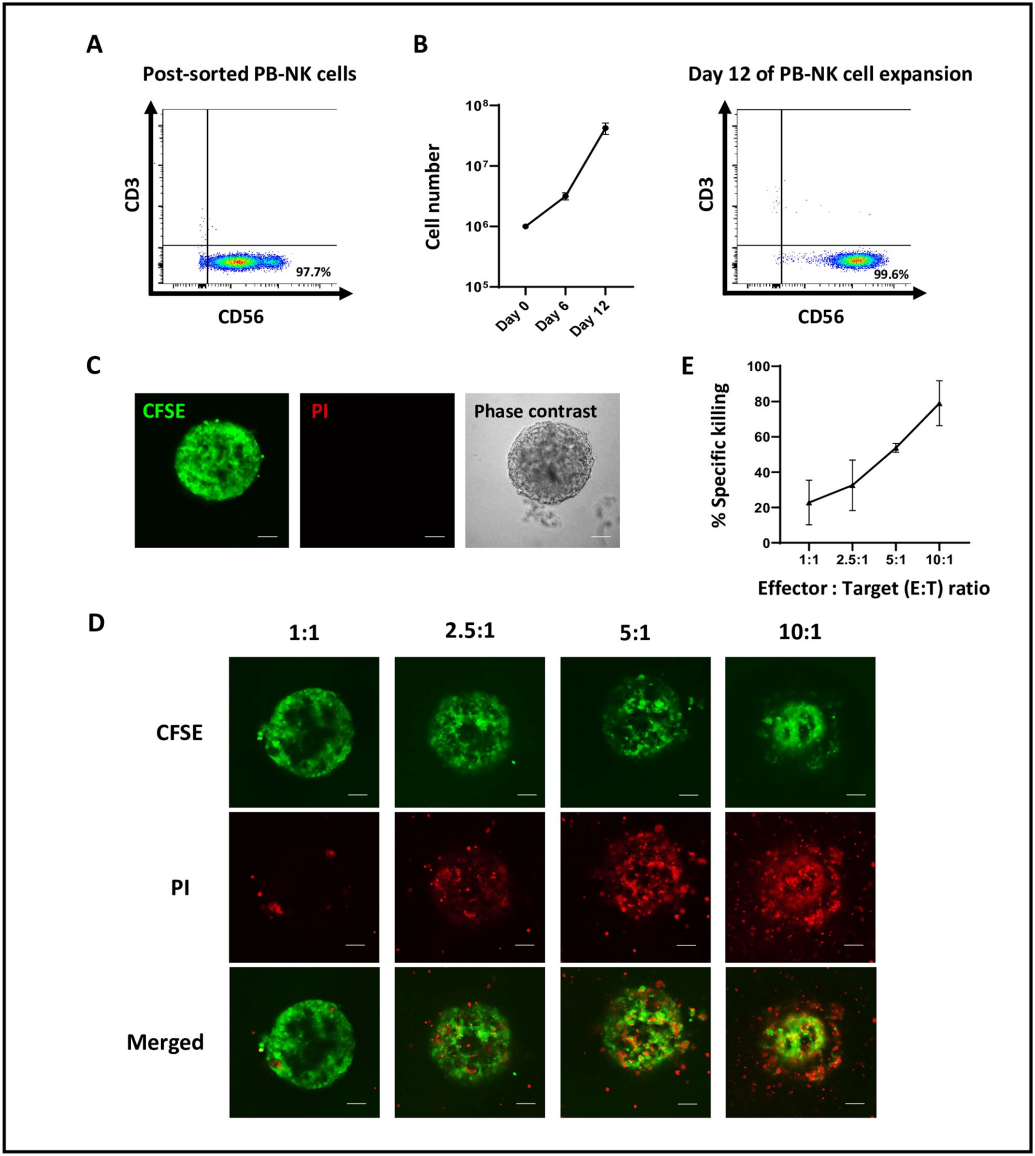
Evaluation of NK cell cytotoxic activity toward 3D tumor spheroids. (A) Flow cytometric analysis of the NK cell-related markers (CD45, CD3, and CD56) of the post-sorted PB-NK cells. (B) Proliferation and flow cytometric analysis of the PB-NK cells in the presence of the genetically modified mIL-21-K562 cell line and hIL-2 (PB-NK cells were isolated from 2 donors, n = 2). (C) Fluorescent and phase-contrast images of the CFSE-labeled KKU213A tumor spheroids in the culture medium containing 2.5% Matrigel^®^ matrix on day 2. Green = tumor cells (CFSE), Red = dead cells (PI), scale bar = 20 μm. (D) Representative fluorescent images of the PB-NK cells and the 3D tumor spheroids at different effector to target ratios (E: T) (1:1, 2.5:1, 5:1, and 10:1). Green = tumor cells (CFSE). Red = dead cells (PI), scale bar = 20 μm. (E) Cytotoxic activity of the PB-NK cells against 3D tumor spheroids at 1:1, 2.5:1, 5:1, and 10:1. The PB-NK cells were obtained from 2 donors. Data are presented as mean ± SEM (n = 2).

PB-NK cells were isolated by negative selection using magnetic beads. The cells were then processed for flow cytometric analysis to ensure that the majority of the post-sorted cells were CD56^+^ NK cells. The isolated cells were 97.7% positive for the NK cell markers (CD45^+^ CD3^-^ CD56^+^) (Fig 2A).

Afterward, NK cells were expanded by co-culturing with the irradiated genetically modified mIL-21-K562 cell line in the presence of hIL-2. Proliferation of NK cells was observed within six days, and the number consistently increased over 12 days. Our protocol could generate 3.4 × 10^7^ NK cells from 1 × 10^6^ cells (34-fold expansion), and on day 12 after expansion, the cells were 99.6% positive for NK cell markers (CD45^+^ CD3^-^ CD56^+^) (Fig 2B).

For the 3D spheroid formation, the KKU213A cell line (obtained from the Japanese Collection of Research Bioresources (JCRB) cell bank, Osaka, Japan), was labeled with 2.5 μM Carboxyfluorescein succinimidyl ester (CFSE), resuspended with 2.5% Matrigel^®^ matrix, and seeded onto the ultra-low attachment round-bottom 96-well plate. The CFSE-labeled KKU213A cell line spontaneously aggregated to form tumor spheroids within two days (Fig 2C). On day 2, the spheroids were co-cultured with PB-NK cells for another three days using four different effector to target (E: T) ratios (1:1, 2.5:1, 5:1, and 10:1) in the presence of 1 μg/ml propidium iodide (PI). On day 5 of the assay, the tumor spheroids were imaged using a confocal microscope, and the cytotoxic activity was calculated using the mean fluorescence intensity (MFI) of PI. In the presence of PB-NK cells, the PI-positive tumor cells within the tumor spheroids can be observed in every E: T ratio (Fig 2D), indicating that the PB-NK cells were capable of inducing cytotoxicity toward the KKU213A tumor spheroids. Notably, the number of PI-positive tumor cells increased with the increasing E: T ratios (Fig 2D). We then evaluated the anti-tumor activity of the PB-NK cells against the 3D tumor spheroids using the MFI of PI. After three days of co-culture, the PB-NK cells exhibited an increase in killing activity in a dose-dependent manner. The killing activity of NK cells reached 79.1 ± 12.7% at 10:1 E: T ratio (Fig 2E). In summary, we have established a simple and feasible protocol to study primary human NK cell cytotoxic activity against 3D tumor spheroids, which can be adapted to other types of tumor and human immune cells to study an *in vitro* cytotoxic activity in a 3D tumor model.

## Supporting information

S1 File Step-by-step protocol, also available on protocol.io. (PDF)

S2 File (PDF)
