# Supplementary material for: A three-dimensional immune-oncology model for studying in vitro primary human NK cell cytotoxic activity
Source: PLoS One. 2022 Mar 21;17(3):e0264366. doi: 10.1371/journal.pone.0264366 (PMC8936498; doi:10.1371/journal.pone.0264366)
Supplement: S1 File — (PDF) [file pone.0264366.s001.pdf]

LAB PROTOCOL

A three-dimensional immune-oncology model for studying *in vitro* primary human NK cell cytotoxic activity.

Nontaphat Thongsin[1,2] and Methichit Wattanapanitch[1*]

[1]Siriraj Center for Regenerative Medicine, Research Department, Faculty of Medicine Siriraj Hospital, Mahidol University, Bangkok, Thailand.

[2]Department of Immunology, Faculty of Medicine Siriraj Hospital, Mahidol University, Bangkok, Thailand

*methichit.wat@mahidol.ac.th

Materials

Isolation of peripheral blood mononuclear cells (PBMCs)

1. 30-ml peripheral blood in vacutainer EDTA blood collection tubes
2. Vacutainer® EDTA blood collection tubes (BD Biosciences, catalog number: 366643)
3. IsoPrep (Robbins Scientific, catalog number: 1070-0-40)
4. Rosewell Park Memorial Institute (RPMI) 1640 (RPMI-1640) medium (Gibco™, catalog number: 31800-022)
5. CryoStor® CS10 Cell Freezing Medium (StemCell™ Technologies, catalog number: 07930)
6. 1 × phosphate-buffered saline (PBS) (Apsalagen, catalog number: AP9000201)
7. 50-ml conical tubes (Wuxi NEST Biotechnology, catalog number: 602052)
8. Hemocytometer (Blaubrand, catalog number: 717810)
9. Slow cooling cryo-container (Corning®, catalog number: 432001)
10. Cryogenic vials (Wuxi NEST Biotechnology, catalog number: 607101)
11. Sterile seropipettes and pipette tips (1000 μl) (KIRGEN®)
12. Transfer pipettes (Wuxi NEST Biotechnology, catalog number 318212)
13. Inverted microscope
14. Tabletop centrifuge
15. Tissue culture hood and humidified incubator (37 °C, 5% CO_2)
16. Liquid nitrogen tank

Negative selection of peripheral blood NK (PB-NK) cells

1. MojoSort™ Human NK cell Isolation Kit (Biolegend, catalog number: 480054)
2. MojoSort™ Magnet (Biolegend, catalog number: 480019)

3. Sorting buffer: 1 × PBS (Apsalagen, catalog number: AP9000201), 0.5% BSA (Sigma-Aldrich, catalog number: 12659-25GM), and 2 mM EDTA (Thermo Fisher Scientific, catalog number: AM9260G)
4. PB-NK culture medium: RPMI-1640 (Gibco™, catalog number: 31800-022), 10% fetal bovine serum (FBS) (Sigma-Aldrich, catalog number: F7524), 2 mM GlutaMAX™ (Gibco™, catalog number: 35050-061), 1% non-essential amino acid (Gibco™, catalog number: 11140-050), 1% penicillin/streptomycin (Gibco™, catalog number: 15140-122), and 100 U/ml hIL-2 (Peprotech, catalog number: 200-02)
5. 25-cm^2 cell culture flask (Wuxi NEST Biotechnology, catalog number: 707003)
6. Hemocytometer (Blaubrand, catalog number: 717810)
7. 5-ml round-bottom tube with cap (Falcon®, catalog number: 352052)
8. 50-ml conical tubes (Wuxi NEST Biotechnology, catalog number: 602052)
9. 70-μm cell strainer (SPL Life Science, catalog number: 93040)
10. Slow cooling cryo-container (Corning®, catalog number: 432001)
11. Cryogenic vials (Wuxi NEST Biotechnology, catalog number: 607101)
12. CryoStor® CS10 Cell Freezing Medium (StemCell™ Technologies, catalog number: 07930)
13. Sterile seropipettes and pipette tips (200 and 1000 μl) (KIRGEN®)
14. Inverted microscope
15. Tabletop centrifuge
16. Vortex
17. Tissue culture hood and humidified incubator (37 °C, 5% CO_2)
18. Liquid nitrogen tank

Expansion of PB-NK cells

1. Irradiated genetically modified membrane-bound IL-21-K562 (mIL-21-K562) cell line
2. mIL-21-K562 cryopreservation medium: 90% FBS (Sigma-Aldrich, catalog number: F7524) and 10% DMSO (Amresco, catalog number: 0231-500ML)
3. PB-NK culture medium: RPMI-1640 (Gibco™, catalog number: 31800-022), 10% FBS (Sigma-Aldrich, catalog number: F7524), 2 mM GlutaMAX™ (Gibco™, catalog number: 35050-061), 1% non-essential amino acid (Gibco™, catalog number: 11140-050), 1% penicillin/streptomycin (Gibco™, catalog number: 15140-122), and 100 U/ml hIL-2 (Peprotech, catalog number: 200-02)
4. 25-cm^2 cell culture flask (Wuxi NEST Biotechnology, catalog number: 707003)
5. Hemocytometer (Blaubrand, catalog number: 717810)
6. Slow cooling cryo-container (Corning®, catalog number: 432001)

7. Cryogenic vials (Wuxi NEST Biotechnology, catalog number: 607101)
8. Sterile seropipettes and pipette tips (200 and 1000 μl) (KIRGEN®)
9. Inverted microscope
10. Tabletop centrifuge
11. Tissue culture hood and humidified incubator (37 °C, 5% CO_2)
12. Liquid nitrogen tank

Evaluation of PB-NK cells using flow cytometry

1. FAC buffer: 3% FBS (Sigma-Aldrich, catalog number: F7524) in 1 × PBS (Apsalagen, catalog number: AP9000201)
2. Fluorescence-conjugated anti-human CD45 (Biolegend, catalog number: 368506), CD3 (Biolegend, catalog number: 300406), CD56 (Biolegend, catalog number: 362509)
3. Zombie Violet™ Fixable Viability Kit (Biolegend, catalog number: 423113)
4. Human AB serum (Sigma-Aldrich, catalog number: H4522-100ML)
5. 1% paraformaldehyde (Sigma-Aldrich, catalog number: P6148-500G)
6. Hemocytometer (Blaubrand, catalog number: 717810)
7. 5-ml round-bottom tube with cap (Falcon®, catalog number: 352052)
8. Sterile seropipettes and pipette tips (200 and 1000 μl) (KIRGEN®)
9. Inverted microscope
10. Tabletop centrifuge
11. Flow cytometer (LSRFortessa™, BD biosciences)

Culture of KKU213A cell line

1. KKU213A culture medium: DMEM/F12 (Gibco™, catalog number: 12500-062), 10% FBS (Sigma-Aldrich, catalog number: F7524), 2 mM GlutaMAX™ (Gibco™, catalog number: 35050-061), and 1% penicillin/streptomycin (Gibco™, catalog number: 15140-122)
2. 0.1% Trypsin-EDTA (Gibco™, catalog number: 25200-072)
3. 1 × PBS (Apsalagen, catalog number: AP9000201)
4. 25-cm^2 cell culture flask (Wuxi NEST Biotechnology, catalog number: 707003)
5. 15-ml conical tubes (Wuxi NEST Biotechnology, catalog number: 601052)
6. Hemocytometer (Blaubrand, catalog number: 717810)
7. Sterile seropipettes and pipette tips (200 and 1000 μl) (KIRGEN®)
8. Inverted microscope
9. Tabletop centrifuge

10. Tissue culture hood and humidified incubator (37 °C, 5% CO_2)

KKU213A labeling using CFSE

1. Carboxyfluorescein succinimidyl ester (CFSE) (Biolegend, catalog number: 423801)
2. KKU213A culture medium: DMEM/F12 (Gibco™, catalog number: 12500-062), 10% FBS (Sigma-Aldrich, catalog number: F7524), 2 mM GlutaMAX™ (Gibco™, catalog number: 35050-061), and 1% penicillin/streptomycin (Gibco™, catalog number: 15140-122)
3. 1 × PBS (Apsalagen, catalog number: AP9000201)
4. Sterile seropipettes and pipette tips (200 and 1000 µl) (KIRGEN®)
5. Hemocytometer (Blaubrand, catalog number: 717810)
6. Tabletop centrifuge
7. Tissue culture hood and humidified incubator (37 °C, 5% CO_2)

Formation of three-dimensional (3D) tumor spheroids

1. CFSE labeled-KKU213A cell line
2. KKU213A culture medium: DMEM/F12 (Gibco™, catalog number: 12500-062), 10% FBS (Sigma-Aldrich, catalog number: F7524), 2 mM GlutaMAX™ (Gibco™, catalog number: 35050-061), and 1% penicillin/streptomycin (Gibco™, catalog number: 15140-122)
3. Matrigel® matrix (Corning®, catalog number: 356234)
4. Ultra-low attachment round-bottom 96-well plate (Corning®, catalog number: 7007)
5. Pre-chilled sterile pipette tips (200 and 1000 µl) (KIRGEN®)
6. Tabletop centrifuge
7. Tissue culture hood and humidified incubator (37 °C, 5% CO_2)
8. Icebox

Cytotoxic activity assay

1. Expanded PB-NK cells
2. PB-NK culture medium: RPMI-1640 (Gibco™, catalog number: 31800-022), 10% FBS (Sigma-Aldrich, catalog number: F7524), 2 mM GlutaMAX™ (Gibco™, catalog number: 35050-061), 1% non-essential amino acid (Gibco™, catalog number: 11140-050), 1% penicillin/streptomycin (Gibco™, catalog number: 15140-122)
3. CFSE labeled-KKU213A tumor spheroid
4. Propidium iodide (PI) (Thermo Fisher Scientific, catalog number: P3566)
5. Sterile seropipettes and pipette tips (200 and 1000 µl) (KIRGEN®)

6. Hemocytometer (Blaubrand, catalog number: 717810)
7. 15-ml conical tubes (Wuxi NEST Biotechnology, catalog number: 601052)
8. Tabletop centrifuge
9. Inverted microscope
10. Confocal microscope (Nikon Instruments Inc., Melville, NY, USA)
11. Tissue culture hood and humidified incubator (37 °C, 5% CO_2)
12. NIS-Elements software

Methods

The following section describes the protocol that we use to evaluate the cytotoxic activity of NK cells toward the 3D-tumor spheroids. The protocol includes the isolation and expansion of PB-NK cells, formation of tumor spheroids, and cytotoxicity assay.

1. Isolation and expansion of peripheral blood NK (PB-NK) cells

1.1 Isolation of PB-NK cells by negative selection using NK cell isolation kit

We isolate peripheral blood mononuclear cells (PBMCs) from 30 ml of peripheral blood collected in vacutainer® EDTA blood collection tubes using density gradient centrifugation.

1. Collect 30 ml of peripheral blood to vacutainer® EDTA blood collection tube and mix the blood thoroughly by inverting.
2. Transfer 15 ml of blood to two 50-ml conical tubes.
3. Add 15 ml of 1× PBS to the blood and mix thoroughly using a sterile serological pipette.
4. Prepare two 50-ml conical tubes containing 9 ml of IsoPrep.
5. Gently overlay 30 ml of diluted blood onto the IsoProp layer using a sterile serological pipette and centrifuge at 800 × g for 30 min, acceleration = 5 and break = off at room temperature.
6. Remove plasma layer using a transfer pipette.
7. Collect the PBMCs to a 50-ml conical tube using a transfer pipette.
8. Wash the cells with 20 ml of 1× PBS and centrifuge at 400 × g for 10 min at room temperature.
9. Repeat the washing step (step 8).
10. After centrifugation, discard the supernatant and resuspend the cell pellet with 10 ml of basal RPMI-1640 medium.
11. Perform cell counting using trypan blue exclusion assay to determine the cell number and viability.
12. Perform PB-NK cell isolation using the MojoSort™ Human NK cell Isolation Kit following the manufacturer's instruction.

13. Resuspend the cell pellet with the PB-NK culture medium and centrifuge the cells at 500 × g for 10 min at room temperature.
14. During centrifugation, perform cell counting using trypan blue exclusion assay to determine the cell number and viability.
15. After centrifugation, resuspend the cell pellet with the PB-NK culture medium and collect 1×10^6 viable cells for PB-NK cell expansion, 4×10^5 viable cells for flow cytometric analysis, and cryopreserve using CryoStor® at a density of 2×10^6 viable cells/ml/vial.

1.2 Expansion of PB-NK cells

Expansion of the PB-NK cells is performed using the genetically modified mIL-21-K562 cell line irradiated with γ-radiation at 10,000 rad. One-day post-irradiation, the mIL-21-K562 cell line is cryopreserved in the mIL-21-K562 cryopreservation medium.

1. Quickly thaw the cryopreserved irradiated mIL-21-K562 cells in a 37 °C water bath until only a small ice crystal remains.
2. Wipe the outside of the cryogenic vial with 70% ethanol.
3. Transfer the cell suspension using a transfer pipette to a 15-ml conical tube containing 9 ml of basal RPMI-1640 medium and take out 10 µl of the cell suspension for counting.
4. Centrifuge the cells at 500 × g for 5 min at room temperature.
5. During centrifugation, perform cell counting using trypan blue exclusion assay to determine the cell number and viability.
6. After centrifugation, discard the supernatant and resuspend the cell pellet with 1 ml of PB-NK culture medium.
7. Co-culture the PB-NK cells (Subheading 1.1, step 15) with the irradiated mIL-21-K562 cells at a 1:2 ratio at a seeding density of 3×10^5 PB-NK cells/ml in the PB-NK culture medium and incubate at 37 °C, 5% CO_2.
8. Change the PB-NK culture medium every three days and add freshly thawed irradiated mIL-21-K562 cells at a 1:1 ratio every six days.

1.3 Evaluation of the sorted PB-NK cells using flow cytometry

We perform flow cytometric analysis to determine the purity of the PB-NK cells after cell sorting and expansion by staining with the common NK cell markers, including CD45, CD3, and CD56. NK cells are identified by $CD45^+$ $CD3^-$ $CD56^+$ population.

1. Centrifuge the cell suspension (Subheading 1.1, step 15) at 500 × g for 5 min at room temperature.

2. Discard the supernatant and block the potential non-specific binding using 100 µl of 10% human AB serum in FAC buffer for 30 min at 4 °C.
3. Add 0.2 µl of Zombie Violet™ Fixable Viability Kit, vortex briefly, and transfer 50 µl of the cell suspension into two 5-ml round-bottom tubes for the NK cell marker and the isotype control staining.
4. Add 1 µl of the fluorescence-conjugated anti-human CD45, CD3, and CD56 or the fluorescence-conjugated isotype control to the cell suspension. Incubate for 15 min in the dark.
5. Add 3 ml of FAC buffer, vortex briefly, and centrifuge at 500 × g for 5 min at room temperature.
6. Discard the supernatant and fix the cells using 500 µl of 1% paraformaldehyde. The stained cells can be stored at 4 °C for up to 2 weeks.
7. Determine the percentage of NK cell population using a flow cytometer (LSRFortessa™) and the FlowJo software. Exclude the dead cells, which stained positive for Zombie Violet™.

2 Formation of three-dimensional (3D) tumor spheroids

For the 3D spheroid formation, the cholangiocarcinoma cell line (KKU213A) is cultured in the medium containing 2.5% Matrigel® matrix on an ultra-low attachment round-bottom 96-well plate for two days.

2.1 KKU213A cell line passaging

Passaging is performed when the cells reach 70-80% confluence, usually 3-4 days, and the cells are seeded onto a 25-cm^2 cell culture flask.

1. Aspirate the culture medium and wash the cells with 3 ml of 1 × PBS.
2. Add 1 ml of 0.1% trypsin-EDTA and incubate the cells at 37 °C for 5 min.
3. After incubation, add 4 ml of DMEM/F-12 medium and resuspend the cells vigorously using a serological pipette.
4. Transfer the cell suspension to a 15-ml conical tube, take out 10 µl of the cell suspension for counting, and centrifuge the cells at 500 × g for 5 min at room temperature.
5. During centrifugation, perform cell counting using the trypan blue exclusion assay to determine the cell number and viability.
6. After centrifugation, discard the supernatant and resuspend the cell pellet with 1 ml culture medium.
7. Transfer 1.5×10^5 viable cells to a 25-cm^2 culture flask containing 5 ml culture medium and incubate at 37 °C, 5% CO_2.

2.2 KKU213A cell line labeling using CFSE

1. Aspirate the culture medium and dissociate the cell using 0.1% trypsin-EDTA (Subheading 2.1, steps 1-6).

2. Transfer 2 × 10^5 viable cells to a 15-ml conical tube, wash with 1 ml of 1 × PBS and centrifuge at 500 × g for 5 min at room temperature.
3. Discard the supernatant and resuspend the cell pellet with 1 ml of 1 × PBS.
4. Add 2.5 µM CFSE solution to the cell suspension, vortex briefly, and incubate at 37 °C, 5% CO_2 for 20 min in the dark.
5. After incubation, add 5 ml of the KKU213A culture medium to the cell suspension and centrifuge at 500 × g for 5 min at room temperature.
6. Discard the supernatant and resuspend the cell pellet with 1 ml of the culture medium.

2.3 Formation of 3D tumor spheroids

1. Prepare the KKU213A culture medium containing 2.5% Matrigel® matrix and chill the medium on ice.
2. Aliquot appropriate cell number of the CFSE-labeled KKU213A cells (Subheading 2.2) to a 15-ml conical tube and centrifuge at 500 × g for 5 min at room temperature.
3. Resuspend the CFSE-labeled KKU213A cells with the pre-chilled KKU213A culture medium containing 2.5% Matrigel® matrix.
4. Seed the CFSE-labeled KKU213A cells onto an ultra-low attachment round-bottom 96-well plate at a seeding density of 2 × 10^3 cells/100 µl culture medium.
5. Centrifuge the 96-well plate at 1,000 × g for 10 min at 4 °C and incubate at 37 °C, 5% CO_2 for two days.
   NOTE: Thaw Matrigel® at 4 °C overnight and always keep Matrigel® under 4 °C until use to prevent gelation. Plasticware that is in contact with Matrigel® should be pre-chilled before use.

3. Cytotoxicity assay

Cytotoxic activity is performed by co-culturing the expanded PB-NK cells with the tumor spheroids at 1:1, 2.5:1, 5:1, and 10:1 (effector: target) ratios for three days. Cytotoxic activity can be determined by the fluorescence intensity of propidium iodide (PI), which stained positive for dead cells, using a confocal microscope.

3.1 Co-culture of PB-NK cells with KKU213A tumor spheroids

1. Change the medium of the PB-NK cells 24 h prior to co-culture.
2. On day 2 of spheroid formation, transfer the PB-NK cells to a 15-ml conical tube, take out 10 µl of the cell suspension for counting, and centrifuge at 500 × g for 5 min at room temperature.
3. During centrifugation, perform cell counting using trypan blue exclusion assay to determine the cell number and viability.

4. After centrifugation, discard the supernatant, resuspend the cell pellet with 1 ml of PB-NK culture medium, and aliquot the appropriate number of PB-NK cells into a 15-ml conical tube for co-culture.
5. Centrifuge at 500 × g for 5 min at room temperature.
6. Discard the supernatant and resuspend the cell pellet with an appropriate volume of PB-NK culture medium, without hIL-2 for co-culture, add 2 μg/ml of propidium iodide (PI) and mix vigorously using a sterile pipette.

   NOTE: The volume of the PB-NK culture medium is 100 μl/well.
7. Add 100 μl of the suspended PB-NK cells to a well of the ultra-low attachment round-bottom 96-well plate.

   NOTE: Gently add the PB-NK cells to avoid disturbing the spheroids. The total volume of culture medium is 200 μl/well, and the final concentration of PI is 1 μg/ml.
8. Centrifuge 96-well plate at 1,000 × g for 10 min at 4 °C.
9. Incubate the cells at 37 °C, 5% CO_2 for three days.

   NOTE: We recommend performing 3 replicates for each ratio. Prepare control wells containing only the tumor spheroids for determining the spontaneous tumor cell death and the tumor spheroids with 0.1% Triton X-100 for determining the maximum cell death.

3.2 Evaluation of cytotoxic activity using a live imaging confocal microscope

1. After three days of co-culture, determine the mean fluorescence intensity (MFI) of PI under a confocal microscope.
2. Cytotoxic activity of the PB-NK cells can be calculated using the following formula:

$$\%\text{Specific killing} = \frac{[(\text{Experimental MFI} - \text{Spontaneous MFI})]}{[(\text{Maximum MFI} - \text{Spontaneous MFI})]} \times 100$$

   The experimental MFI represents the mean fluorescence intensity of PI in the presence of effectors at a given effector to target (E: T) ratio, whereas the spontaneous MFI represents the mean fluorescence intensity of PI in the absence of effector cells. Maximum MFI represents the mean fluorescence intensity of PI from tumor spheroids that are treated with 0.1% Triton™ X-100.
