# Supplementary material for: A three-dimensional immune-oncology model for studying in vitro primary human NK cell cytotoxic activity
Source: PLoS One. 2022 Mar 21;17(3):e0264366. doi: 10.1371/journal.pone.0264366 (PMC8936498; doi:10.1371/journal.pone.0264366)
Supplement: S2 File — (PDF) [file pone.0264366.s002.pdf]

Supplementary file 2

Table 1: Expansion of PB-NK cells using irradiated genetically modified mIL-21-K562 cell line.

	**Cell number**		
	**Day 0**	**Day 6**	**Day 12**
**Donor 1**	1,000,000	2,866,666	35,905,000
**Donor 2**	1,000,000	3,454,545	48,640,000

Table 2: *In vitro* cytotoxicity of the PB-NK cells against KKU213A tumor spheroids at 72 h.

	**% Specific killing**			
	**1:1**	**2.5:1**	**5:1**	**10:1**
**Donor 1**	35.44	46.89	56.30	91.78
**Donor 2**	10.18	18.33	51.33	66.34
